# Identification of the O/ME-SA/Ind-2001e Sublineage of Foot-and-Mouth Disease Virus in Cambodia

**DOI:** 10.3389/fvets.2021.749966

**Published:** 2021-10-29

**Authors:** Soyoon Ryoo, HyunJi Lee, Da-Rae Lim, Jung-Won Lee, Seng Bunnary, Sothyra Tum, Dong Sook Lee, Hyeonwoo Hwang, SomGyeol Jeong, JinJu Nah, Bok Kyung Ku, Jae-Myung Kim, Sang-Ho Cha

**Affiliations:** ^1^Foot-and-Mouth Disease Research Division, Animal and Plant Quarantine Agency, Gimcheon-si, South Korea; ^2^Department of Animal and Production, National Animal Health and Production Research Institute, Phnom Penh, Cambodia

**Keywords:** foot-and-mouth disease virus, ME-SA, Ind-2001e, Cambodia, transboundary movement

## Abstract

Foot-and-mouth (FMD) is endemic in Cambodia with numerous outbreaks in cattle, pigs and other susceptible animal species reported every year. Historically, these outbreaks were caused by the FMD virus (FMDV) of serotype O PanAsia and Mya-98 lineages and serotype A Sea-97 lineage. However, the trans-pool movement of FMDV between inter-pool regions or countries throughout FMD endemic regions has raised concerns regarding infection with the new genotype or serotype of FMDV in Cambodia. In this study, 19 sequences of VP1 coding region obtained from 33 clinical samples collected from FMDV-affected cattle farms in Cambodia during January to March 2019 were genetically characterized to identify the genotypes/lineages of FMDV.

Phylogenetic analysis of VP1 coding sequences revealed that recent field viruses belonged to O/ME-SA/Ind-2001e (15.8%), O/ME-SA/PanAsia (52.7%), and A/ASIA/Sea-97 (31.5%). Besides, the field viruses of O/ME-SA/Ind-2001e in Cambodia showed 93.5–96.8% identity with the VP1 coding sequences of the same sublineage viruses from pool 1 and 2 surrounding Cambodia. This is the first report of O/ME-SA/Ind-2001e infection in Cambodia, suggesting that the trans-pool movement of the new genotype should be closely monitored for efficient control of FMD.

## Introduction

Foot-and-mouth disease (FMD) is a highly contagious vesicular disease that affects cloven-hoofed animals and is caused by the FMD virus (FMDV). FMDV is divided into seven immunologically distinct serotypes [O, A, C, Asia 1, and Southern African Territories (1–3)], which are also composed of various topotypes and lineages (1).

Endemic circulation of FMDVs by distinct viral strains has been reported to form seven regional pools that independently undergo evolution (2). Serotypes O, A, and Asia 1 have been identified in Pool 1, spreading across the South East Asian countries comprising of Cambodia, Lao People's Democratic Republic, Myanmar, Peninsular Malaysia, Thailand and Vietnam. Among the serotypes in the recently reported FMD outbreaks, serotype O is the most prevalent, with two distinct topotypes (Southeast Asia and Middle East-South Asia) in the region; three lineages (O/ME-SA/Ind-2001, O/ME-SA/PanAsia, and O/SEA/Mya-98) of Middle East-South Asia (ME-SA) and SEA topotypes are the major causatives of FMD (3). Serotype A is also prevalent in the region with a main lineage of A/ASIA/Sea97 (3).

The ME-SA topotype was reported to emerge independently throughout South Asia and the Middle East, and three lineages (PanAsia, PanAsia-2, and Ind-2001) of this topotype have been identified in recent years. The O/ME-SA/Ind-2001 lineage was classified into five sublineages, *a–e* (4). Extensive monitoring of this topotype revealed trans-pool movement of the lineage O/ME-SA/Ind-2001 throughout the Middle East, North Africa, and Southeast Asia (Lao People's Democratic Republic, Vietnam, Myanmar, etc.), which has been occurring since 2009 (4–7).

In Cambodia, two serotypes (serotypes A and O) of FMDV have caused endemic FMD outbreaks.

The serotype O FMDV lineages circulating in Cambodia were identified as O/SEA/Cam-94 lineages in 1994 and 1998 (8) and O/ME-SA/PanAsia first reported in 2000, and only O/ME-SA/PanAsia has been reported before 2019 (3).

In this study, we obtained clinical samples from FMD outbreaks in January through March 2019 in Cambodia. Since FMD is endemic in Cambodia and there are reports of the incursion of new lineages of viruses into South East Asia, we aimed to identify genotype of the viruses causing the FMD outbreaks, while monitoring possible emergence of new lineages/ sublineages that would have epidemiological importance.

## Materials and Methods

### Clinical Samples and Virus Isolation

Cambodian veterinary authorities were notified through their field-based veterinarians about all FMD outbreaks across Cambodia in January through March, 2019. Samples were collected during FMD outbreaks from different households. The collected samples were stored in viral transport media (BD Diagnostics) and sent to National Animal Health and Production Research Institute (NAHPRI, Cambodia) in icebox and stored at −80°C until shipment to the Animal and Plant Quarantine Agency (APQA, South Korea). Thirty-three clinical samples (epithelium and saliva) collected from suspected FMD cases in Cambodia were shipped on dry-ice under international regulations from the NAHPRI to the APQA.

LFBK-α_V_β_6_ cell lines, supplied by the Plum Island Animal Disease Center (Long Island, NY, USA), were cultured in growth media composed of Dulbecco's Modified Eagle Medium (DMEM, Corning, NY, USA), 1× Antibiotic-Antimycotic solutions (Corning, NY, USA), and 10% fetal bovine serum (Corning, NY, USA) for viral isolation (9). For viral isolation, 20% tissue homogenates were prepared in DMEM media (Corning, NY, USA) containing 1× Antibiotic-Antimycotic solutions (Corning, NY, USA) and inoculated onto LFBK- α_V_β_6_ cell lines prepared 1 day before inoculation. When a 90% cytopathic effect was observed, the cells and culture media were harvested and stored at 80°C until analysis. If no CPE was observed, the cells were frozen and thawed, and the supernatant was used to be inoculated onto new fresh cells, which were examined for CPE for another 48 h.

### 3D Real-Time RT-PCR and Sequencing of VP1 Coding Region

Viral RNA was extracted from clinical samples and cell-isolated viruses using the MagnaPure96 system (Roche, Basel, Switzerland) and Viral NA small Volume kit (Cat No. 06 543 588 001). The 3D real-time reverse transcription (RT)-polymerase chain reaction (PCR) assay was conducted as described previously (10) using a standard method for the molecular diagnosis of FMD of the clinical samples (*n* = 33).

The VP1 coding region (1D) was amplified along with the flanking regions of the 1C and 2A regions by RT-PCR according to published protocols (11). Each amplicon (820 bp) was sequenced using the Big Dye Terminator v3.1 Cycle sequencing kit (Thermo Fisher Scientific, Waltham, MA, USA) with the same primers as used for RT-PCR on an ABI 3,730 DNA analyzer (Applied Biosystems, Foster City, CA, USA).

### Nucleotide/Amino Acid Sequence and Phylogenetic Analysis

VP1 coding sequences (636 bp) were aligned using BioEdit (12), and nucleotide (NT) or amino acid (AA) sequence homology was analyzed among the viral isolates. The sequences were subjected to evolutionary analyses using MEGAX software (13). A phylogenetic tree was constructed by the maximum likelihood method based on the generalized time-reversible model (14) using the VP1 coding sequences of Cambodian field viruses (*n* = 19) and other field isolates from the GenBank database. The percentage of replicate trees in which associated taxa clustered together in the bootstrap test (1,000 replicates) is shown next to the branches (15). The “partial deletion” option was used for gaps/missing data.

## Results

### FMD Outbreaks and Diagnosis

According to Cambodian veterinary authorities, 33 FMD outbreaks were reported in two provinces (Kampong Speu and Prey Veng) in January through March, 2019, affecting cattle with typical FMD clinical signs, including lameness; sharply decreased milk production; and ruptured vesicles on the tongue, teat, and dental pad along with erosions and inflammation of the interdigital space. Clinical samples were obtained from the tongues, vesicular tissues, and lips of FMD-affected cattle, and 3D real-time RT-PCR confirmed 29 FMD outbreaks in Kampong Speu (*n* = 21) and Prey Veng (*n* = 8) provinces (Table 1).

**Table 1:** Outbreaks and diagnosis FMD in Cambodia (January–March 2019).

**Sample ID**	**Collected date**	**Province**	**Spieces**	**Sample type**	**Ct value**	**Virus isolation**	**Lineages**	**Strain ID**	**Accession no**.
CAM 1	Jan. 21	Kampong Speu	Cattle	Epithelium and Saliva	30.12	–			
CAM 2	Jan. 21	Kampong Speu	Cattle	Epithelium and Saliva	19.38	+	O/ME-SA/PanAsia	O/CAM 2/2019	OK107516
CAM 3	Jan. 21	Kampong Speu	Cattle	Epithelium and Saliva	21.56	+	O/ME-SA/PanAsia	O/CAM 3/2019	OK107517
CAM 4	Jan. 21	Kampong Speu	Cattle	Epithelium and Saliva	23.81	+	O/ME-SA/PanAsia	O/CAM 4/2019	OK107518
CAM 5	Jan. 21	Kampong Speu	Cattle	Epithelium and Saliva	26.57	+	O/ME-SA/PanAsia	O/CAM 5/2019	OK107519
CAM 6	Jan. 22	Kampong Speu	Cattle	Epithelium	32.58	–			
CAM 7	Jan. 22	Kampong Speu	Cattle	Epithelium	22.24	+	O/ME-SA/PanAsia	O/CAM 7/2019	OK107520
CAM 8	Jan. 22	Kampong Speu	Cattle	Epithelium and Saliva	21.72	+	O/ME-SA/PanAsia	O/CAM 8/2019	OK107521
CAM 9	Jan. 22	Kampong Speu	Cattle	Epithelium	21.08	+	O/ME-SA/PanAsia	O/CAM 9/2019	OK107522
CAM 10	Jan. 22	Kampong Speu	Cattle	Epithelium	27.95	–			
CAM 11	Jan. 27	Kampong Speu	Cattle	Epithelium	25.28	+	O/ME-SA/PanAsia	O/CAM 11/2019	OK107523
CAM 12	Jan. 27	Kampong Speu	Cattle	Epithelium	24.57	+	O/ME-SA/PanAsia	O/CAM 12/2019	OK107524
CAM 13	Jan. 27	Kampong Speu	Cattle	Epithelium	25.17	+	O/ME-SA/PanAsia	O/CAM 13/2019	OK107525
CAM 14	Jan. 27	Kampong Speu	Cattle	Epithelium	N/A	–			
CAM 15	Jan. 27	Kampong Speu	Cattle	Epithelium	N/A	–			
CAM 16	Mar. 2	Prey veng	Cattle	Epithelium	29.99	+	A/ASIA/Sea-97	A/CAM 16/2019	
CAM 17	Mar. 2	Prey veng	Cattle	Epithelium	36.33	–			
CAM 18	Mar. 2	Prey veng	Cattle	Epithelium	35.81	–			
CAM 19	Mar. 2	Prey veng	Cattle	Epithelium	29.02	+	A/ASIA/Sea-97	A/CAM 19/2019	
CAM 20	Mar. 2	Prey veng	Cattle	Epithelium	22.26	+	A/ASIA/Sea-97	A/CAM 20/2019	
CAM 21	Jan. 21	Kampong Speu	Cattle	Epithelium and Saliva	N/A	–			
CAM 22	Jan. 21	Kampong Speu	Cattle	Epithelium and Saliva	21.18	+	O/ME-SA/Ind-2001e	O/CAM 22/2019	MZ634454
CAM 23	Jan. 21	Kampong Speu	Cattle	Epithelium and Saliva	32.08	–			
CAM 24	Jan. 21	Kampong Speu	Cattle	Epithelium and Saliva	31.48	–			
CAM 25	Jan. 21	Kampong Speu	Cattle	Epithelium and Saliva	37.17	–			
CAM 26	Jan. 22	Kampong Speu	Cattle	Epithelium	25.48	–			
CAM 27	Jan. 22	Kampong Speu	Cattle	Epithelium	35.24	–			
CAM 28	Jan. 22	Kampong Speu	Cattle	Epithelium and Saliva	N/A	–			
CAM 29	Jan. 22	Kampong Speu	Cattle	Epithelium	31.84	+	O/ME-SA/Ind-2001e	O/CAM 29/2019	MZ634455
CAM 30	Jan. 22	Kampong Speu	Cattle	Epithelium	31.56	+	O/ME-SA/Ind-2001e	O/CAM 30/2019	MZ634456
CAM 44	Jan. 15	Prey veng	Cattle	Epithelium and Saliva	21.19	+	A/ASIA/Sea-97	A/CAM 44/2019	
CAM 45	Feb. 15	Prey veng	Cattle	Epithelium and Saliva	21.33	+	A/ASIA/Sea-97	A/CAM 45/2019	
CAM 46	Mar. 15	Prey veng	Cattle	Saliva	16.13	+	A/ASIA/Sea-97	A/CAM 46/2019	

### Viral Isolation and Sequencing of VP1 Coding Region

Out of 33 samples, 29 numbers were positive for 3D real-time RT-PCR (16.13–37.17 Ct value range).

Among 33 clinical samples subjected to virus isolation, 19 clinical samples showed cytopathic effect and the viral isolation was highly successful (15/16, 93.7%) under the Ct value 27. VP1 coding region sequencing of all viral isolates (*n* = 19) was completed and there was no insertion or deletion of any nucleotide on the region. The VP1 coding sequences of O/ME-SA/Ind-2001e and O/ME-SA/PanAsia were submitted to the GenBank database under accession number MZ634454-634456, OK107516-107525, respectively (Table 1).

### Sequence Homology and Phylogenetic Analysis

**Figure 1:**
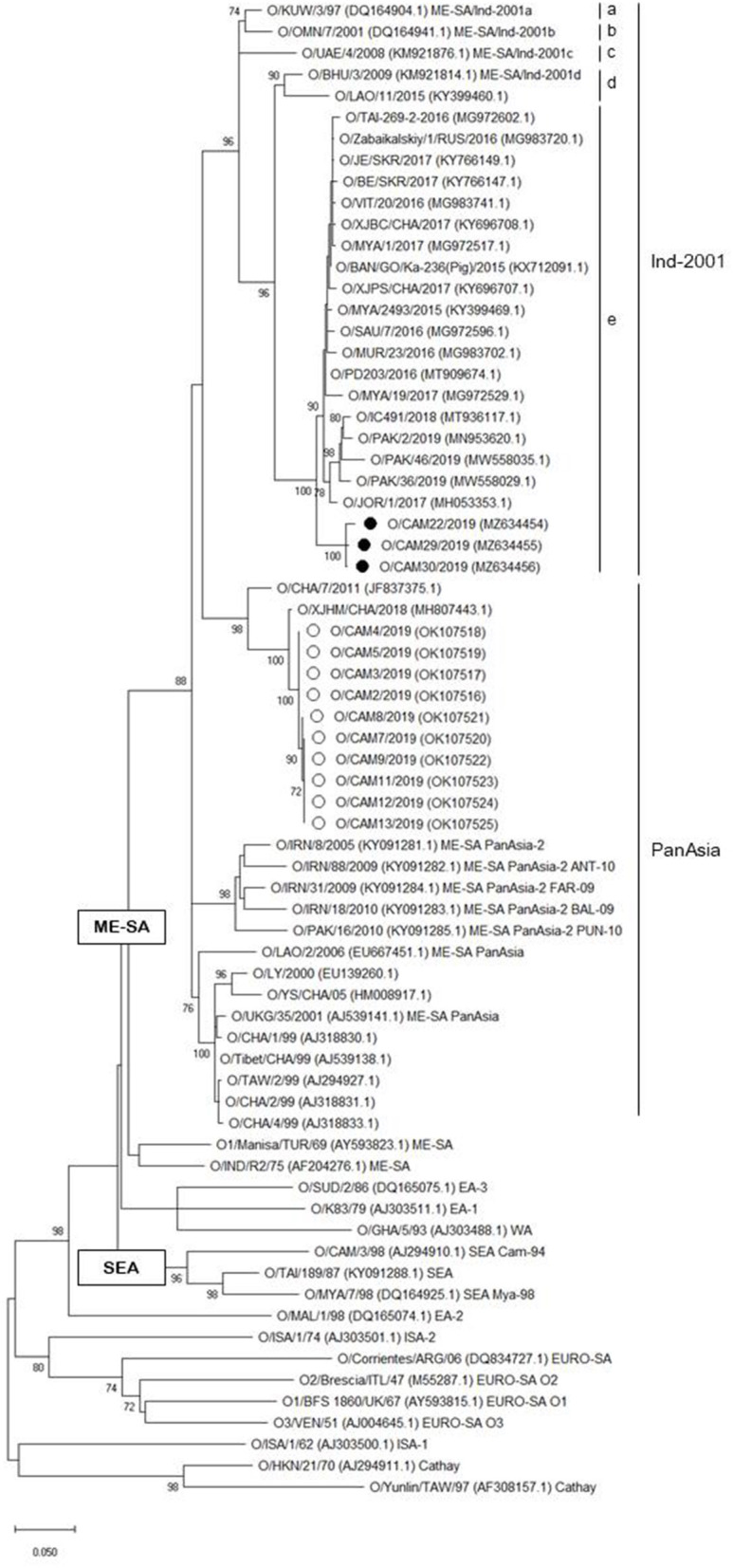
Maximum likelihood tree showing the relationship of the VP1 coding sequence of O/ME-SA/Ind-2001e (black filled circle) and O/ME-SA/PanAsia (open circle) from Cambodia to other FMD type O viruses. The percentages of replicate trees in which the associated taxa clustered together in the bootstrap test (1,000 replicates) are shown next to the branches%.

A phylogenetic tree was constructed using nucleotide sequence (636 bp) of the VP1 coding region of the Cambodian field isolates, including reference FMDV isolates originating from countries surrounding Cambodia. The phylogenetic analysis indicated that the FMD viral isolates were classified as lineages O/ME-SA/PanAsia (*n* = 10), O/ME-SA/Ind-2001e (*n* = 3), and A/ASIA/Sea-97 (*n* = 6). Two lineages, O/ME-SA/PanAsia and O/ME-SA/Ind-2001e, were isolated from Kampong Speu, whereas A/AISA/Sea-97 was isolated from Prey Veng (Table 1). Besides, the field isolates of O/ME-SA/Ind-2001e were grouped with all field isolates belonging to O/ME-SA/Ind-2001e from the Pool 1 and Pool 2 regions and West Asia (Figure 1).

The 1D region varied from 98.8 (100) to 99.5% (100%) among Cambodian O/ME-SA/Ind-2001e field isolates and the Cambodian O/ME-SA/Ind-2001e isolates showed 93.5–96.8% identity with the VP1 coding sequences of the same sublineage viruses in Pool 1 and 2 (Figure 1). Among the reference strains examined, O/PD203/2016 (MT909674.1) of an Indian isolate showed the highest NT and AA sequence identities of 96.8% and 100%, respectively, to O/CAM30/2019 (MZ634456.1), with a range of NT (AA) sequence identity of 96.2% (100%)-−96.8% (100%) among the Cambodian isolates. O/MYA/1/2017 (Myanmar, MG972517.1) also showed close genetic relatedness to the Cambodian isolate (O/CAM30/2019) with 96.6% (100%) NT (AA) sequence identity.

## Discussion

The serotypes O, A, and Asia 1 FMDVs have been reported as endemic to the Pool 1 and Pool 2 regions (3). FMDV spreads between pool regions or countries by wild animals and active transboundary movement of live livestock or livestock product, leading to emerging or re-emerging novel genotypes (16). Cambodia, in the Pool 1 region, has been affected by transboundary spread of the disease from neighboring countries. Even if there may be a circulation of O/SEA/Cam-94 lineage in a certain time of 1990's little information was documented for the lineage (8). The serotype O PanAsia lineage circulating in Cambodia before 2019 was first reported in 2000 and may have been introduced through infected animals or animal products from Vietnam (3, 17), whereas serotype A Sea-97 FMDV infection dates back to 2006 and was introduced *via* the southern provinces from Thailand (18). With an unknown origin of serotype Asia 1 infection in 1997, the three serotypes of FMDV have been circulating in animal populations in Cambodia (18). Therefore, a sudden emergence of novel sublineages in Cambodia is plausible under currently active trans-boundary movement of novel FMDV sublineages.

The clinical samples were collected from cattle showing typical FMD clinical signs and pathological lesions from households in two southern provinces (Kampong Speu and Prey Veng), where FMD outbreaks were reported by field veterinarians between January and March 2019. Sequencing of VP1 coding region revealed that O/ME-SA/PanAsia and A/ASIA/Sea-97 were the main lineages (>85% prevalence) infected in animals in the regions. Importantly, the first detection of the O/ME-SA/Ind-2001e lineage in Cambodia was made in samples collected from Kampong Speu. However, it was unfortunate that comparison of VP1 coding sequences with the same lineages circulating in neighboring countries could not specify origin of the novel genotype, O/ME-SA/Ind-2001e, of Cambodian isolates due to limited VP1 coding sequence information originated from countries surrounding Cambodia and no thorough epidemiological studies on FMD outbreaks caused by the novel genotype, even if some isolates were close to Cambodian field isolates of the O/ME-SA/Ind-2001e. Nevertheless, it is assumed that there is still a possibility of introduction from neighboring countries in the same pool region with the similar epidemiological history to the emergence of serotype O and A FMD viruses in Cambodia.

Even if the O/ME-SA/Ind-2001e sublineage caused FMD outbreaks with 15% prevalence, this sublineage may become one of the main genotypes in Cambodia based on the following assumptions. First, field viruses investigated in this study were isolated from FMD outbreaks in a short period of time (the first 3 months of 2019); second, FMD has tended rapidly to spread and become endemic through livestock of traditional husbandry system by small holder farmers in Cambodia (19) and third, mixed infection among different lineages (PanAsia and Ind-2001) under the same serotype was observed in the same province (Kampong Speu). Mixed infections of viruses with different genotypes among animal populations are often observed among viruses with low cross-reactivity (20).

In this study, FMD field viruses of O/ME-SA/Ind-2001e were first detected in Cambodia from early 2019 FMD outbreaks. Further genetic analysis using recent field isolates from countries of pool regions surrounding Cambodia are required to define trans-movement of the novel sublineage to Cambodia in the future. Besides, close monitoring of FMD outbreaks and the spread of O/ME-SA/Ind-2001e in Cambodia should be performed to evaluate nationwide distributions and to establish efficient control strategies against the novel sublineage.

## Data Availability Statement

The original contributions presented in the study are included in the article, further inquiries can be directed to the corresponding author.

## Ethics Statement

No ethical approval was required for this study as sample collection was performed using standard diagnostic procedures with no harm to the animals. Written informed consent was obtained from the owners for the participation of their animals in this study.

## Author Contributions

SR and SHC contributed to the conception and design of the study. HL performed the sequencing studies and the phylogenetic analysis. JWL, HH, and SJ contributed to isolate viruses from samples. SB and ST coordinated sample collection and shipment. SR wrote the first draft. DRL, DSL, JN, and BKK helped write drafts of the manuscript and assisted with analysis. JMK directed the project. All authors contributed to manuscript revision and read and approved the submitted version.

## Funding

This research was supported by the research grants from the Animal and Plant Quarantine Agency (Project No. I-1543082-2018-22-02).

## Conflict of Interest

The authors declare that the research was conducted in the absence of any commercial or financial relationships that could be construed as a potential conflict of interest.

## Publisher's Note

All claims expressed in this article are solely those of the authors and do not necessarily represent those of their affiliated organizations, or those of the publisher, the editors and the reviewers. Any product that may be evaluated in this article, or claim that may be made by its manufacturer, is not guaranteed or endorsed by the publisher.

## References

[1] Samuel AR Knowles NJ. Foot-and-mouth disease type O viruses exhibit genetically and geographically distinct evolutionary lineages (topotypes). J Gen Virol. (2001) 82:609–21. 10.1099/0022-1317-82-3-609 11172103

[2] Paton DJ Sumption KJ Charleston B. Options for control of foot-and-mouth disease: knowledge, capability and policy. Philos Trans R Soc Lond B Biol Sci. (2009) 364:2657–67. 10.1098/rstb.2009.0100 19687036 PMC2865093

[3] Blacksell SD Siengsanan-Lamont J Kamolsiripichaiporn S Gleeson LJ Windsor PA. A history of FMD research and control programmes in Southeast Asia: lessons from the past informing the future. Epidemiol Infect. (2019) 147:e171. 10.1017/S0950268819000578 31063108 PMC6499730

[4] Bachanek-Bankowska K Di Nardo A Wadsworth J Mioulet V Pezzoni G Grazioli S. Reconstructing the evolutionary history of pandemic foot-and-mouth disease viruses: the impact of recombination within the emerging O/ME-SA/Ind-2001 lineage. Sci Rep. (2018) 8:14693. 10.1038/s41598-018-32693-8 30279570 PMC6168464

[5] Jamal SM Khan S Knowles NJ Wadsworth J Hicks HM Mioulet V. Foot-and-mouth disease viruses of the O/ME-SA/Ind-2001e sublineage in Pakistan. Transbound Emerg Dis. (2021). 10.1111/tbed.14134 33915027

[6] Knowles NJ Bachanek-Bankowska K Wadsworth J Mioulet V Valdazo-Gonzalez B Eldaghayes IM. Outbreaks of foot-and-mouth disease in Libya and Saudi Arabia during 2013 due to an exotic O/ME-SA/Ind-2001 lineage virus. Transbound Emerg Dis. (2016) 63:e431–5. 10.1111/tbed.12299 25483996

[7] Qiu Y Abila R Rodtian P King DP Knowles NJ Ngo LT. Emergence of an exotic strain of serotype O foot-and-mouth disease virus O/ME-SA/Ind-2001d in South-East Asia in 2015. Transbound Emerg Dis. (2018) 65:e104–e12. 10.1111/tbed.12687 28856846

[8] Knowles NJ Davies PR Henry T O'Donnell V Pacheco JM Mason PW. Emergence in Asia of foot-and-mouth disease viruses with altered host range: characterization of alterations in the 3A protein. J Virol. (2001) 75:1551–6. 10.1128/JVI.75.3.1551-1556.2001 11152528 PMC114061

[9] Gray AR Wood BA Henry E Azhar M King DP Mioulet V. Evaluation of cell lines for the isolation of foot-and-mouth disease virus and other viruses causing vesicular disease. Front Vet Sci. (2020) 7:426. 10.3389/fvets.2020.00426 32851014 PMC7401924

[10] Callahan JD Brown F Osorio FA Sur JH Kramer E Long GW. Use of a portable real-time reverse transcriptase-polymerase chain reaction assay for rapid detection of foot-and-mouth disease virus. J Am Vet Med Assoc. (2002) 220:1636–42. 10.2460/javma.2002.220.1636 12051502

[11] Le VP Lee KN Nguyen T Kim SM Cho IS Khang DD. A rapid molecular strategy for early detection and characterization of Vietnamese foot-and-mouth disease virus serotypes O, A, and Asia 1. J Virol Methods. (2012) 180:1–6. 10.1016/j.jviromet.2011.11.028 22172973

[12] Hall T Biosciences I Carlsbad C. BioEdit: an important software for molecular biology. GERF Bull Biosci. (2011) 2:60–1.

[13] Kumar S Stecher G Li M Knyaz C Tamura K MEGA X. Molecular evolutionary genetics analysis across computing platforms. Mol Biol Evol. (2018) 35:1547–9. 10.1093/molbev/msy096 29722887 PMC5967553

[14] Nei M Kumar S. Molecular Evolution and Phylogenetics. Oxford: Oxford University Press (2000).

[15] Felsenstein J. Confidence limits on phylogenies: an approach using the bootstrap. Evolution. (1985) 39:783–91. 10.1111/j.1558-5646.1985.tb00420.x 28561359

[16] Bertram MR Bravo de Rueda C Garabed R Dickmu Jumbo S Moritz M Pauszek S. Molecular epidemiology of foot-and-mouth disease virus in the context of transboundary animal movement in the far North Region of Cameroon. Front Vet Sci. (2018) 5:320. 10.3389/fvets.2018.00320 30619901 PMC6301994

[17] Gleeson LJ. A review of the status of foot and mouth disease in South-East Asia and approaches to control and eradication. Rev Sci Tech. (2002) 21:465–75. 10.20506/rst.21.3.1346 12530354

[18] Tum S Robertson ID Edwards J Abila R Morzaria S. Seroprevalence of foot-and-mouth disease in the southern provinces of Cambodia. Trop Anim Health Prod. (2015) 47:541–7. 10.1007/s11250-015-0760-4 25616981

[19] Sieng S Patrick IW Windsor PA Walkden-Brown SW Sar C Smith RGB. Knowledge, attitudes and practices of smallholder farmers on foot and mouth disease control in two Cambodian provinces. Transbound Emerg Dis. (2021). 10.22541/au.161554875.53061080/v1 34105252

[20] Al-Hosary AA Kandeil A El-Taweel AN Nordengrahn A Merza M Badra R. Co-infection with different serotypes of FMDV in vaccinated cattle in Southern Egypt. Virus Genes. (2019) 55:304–13. 10.1007/s11262-019-01645-3 30771081

